# An efficient root transformation system for CRISPR/Cas9-based analyses of shoot–root communication in cucurbit crops

**DOI:** 10.1093/hr/uhab082

**Published:** 2022-01-20

**Authors:** Shouyu Geng, Hamza Sohail, Haishun Cao, Jingyu Sun, Zhi Chen, Lijian Zhou, Wenbo Wang, Runwen Ye, Li Yang, Zhilong Bie

**Affiliations:** 1 Key Laboratory of Horticultural Plant Biology, Ministry of Education, College of Horticulture and Forestry Sciences, Huazhong Agricultural University, Wuhan 430070, China; 2 Institute of Facility Agriculture, Guangdong Academy of Agricultural Sciences, Guangzhou 510640, China

## Abstract

Cucurbit crops are suitable models for studying long-distance signaling in horticultural plants. Although thousands of substances are graft transmissible in cucurbits, functional studies have been hampered by the lack of efficient genetic transformation systems. Here, we report a convenient and efficient root transformation method for several cucurbit crops that will facilitate studies of functional genes and shoot–root crosstalk. We obtained healthy plants with completely transformed roots and non-transgenic shoots within 6 weeks. Furthermore, we combined this root transformation method with grafting, which allowed for gene manipulation in the rootstock. We validated our system by exploring salt tolerance mechanisms using a cucumber (*Cucumis sativus*)/pumpkin (*Cucurbita moschata* Duch*.*) (scion/rootstock) graft in which the sodium transporter gene *High-affinity K^+^ transporter1* (*CmoHKT1;1*) was edited in the pumpkin rootstock and by overexpressing the pumpkin tonoplast Na^+^/H^+^ antiporter gene *Sodium hydrogen exchanger4* (*CmoNHX4*) in cucumber roots.

## Introduction

Cucurbits are economically important crops because of their dietary and medicinal value [1]. Grafting of a high-yield scion onto a stress-resistant rootstock has been used to improve crop production for thousands of years, and grafting is also an ideal approach for exploring shoot–root crosstalk in plants [2–4]. Grafting among the different genera of Cucurbitaceae is straightforward, and their phloem and xylem saps can be easily obtained; therefore, cucurbit crops are excellent models for studying long-distance signaling and graft-transmissible signals [5–7]. However, the lack of an efficient transgene system in these crops has limited long-distance signaling studies. Although stable transformation systems have been established in watermelon (*Citrullus lanatus*) and cucumber (*Cucumis sativus*) [8, 9], they are lacking in other cucurbit species, such as pumpkin (*Cucurbita moschata* Duch*.*), melon (*Cucumis melo*), bottle gourd (*Lagenaria siceraria*), and luffa gourd (*Luffa acutangula*). *Agrobacterium rhizogenes*–mediated root transformation is a rapid and convenient alternative to conventional stable transformation procedures for gene manipulation *in vivo* [10, 11]. Moreover, the genetic modification of rootstock roots could offer a convenient way to investigate crosstalk between rootstocks and scions. Nonetheless, an efficient *A. rhizogenes*–mediated root transformation system has not been established for most cucurbit crops.

Grafting is a practical approach for overcoming soil-borne diseases and increasing abiotic stress resistance in crops [12]. Pumpkin is the most widely used rootstock for cucurbit crops [7, 12, 13]. For instance, grafting onto pumpkin rootstock has been used to increase the salt tolerance of a cucumber scion. In that case, the mechanism of enhanced salt tolerance was related to the higher capacity of pumpkin roots to limit the long-distance transport of Na^+^ to shoots compared to that of cucumber roots [13]. X-ray analysis revealed that much more Na^+^ is sequestered in the cortex of pumpkin roots than in cucumber roots, suggesting that the transport of Na^+^ to the stele is restricted; thus, its long-distance transport to shoots is limited [14]. The higher salt tolerance of pumpkin is also associated with a higher K^+^ uptake capacity relative to that of cucumber [5]. However, the molecular mechanism and the genes responsible for these differences between pumpkin and cucumber are largely unknown.

Here, we developed a convenient, efficient, and rapid root transformation system for pumpkin and several other cucurbit crops. Healthy plants with roots that had been completely transformed were obtained within 6 weeks. This root transformation system was combined with grafting to take advantage of gene manipulation in the rootstock. We demonstrated the feasibility of our method using the clustered regularly interspaced short palindromic repeats (CRISPR)/CRISPR-associated protein 9 (Cas9) genome editing technology to knock out the sodium transporter gene *CmoHKT1;1* in the pumpkin rootstock of cucumber/pumpkin grafted plants and by overexpressing the pumpkin tonoplast Na^+^/H^+^ antiporter gene *CmoNHX4* in cucumber roots. Our results demonstrate that this robust root transformation system is efficient for functional analysis of genes in the roots of cucurbit crops and also provides a promising approach for studying shoot–root communication.

**Figure 1:**
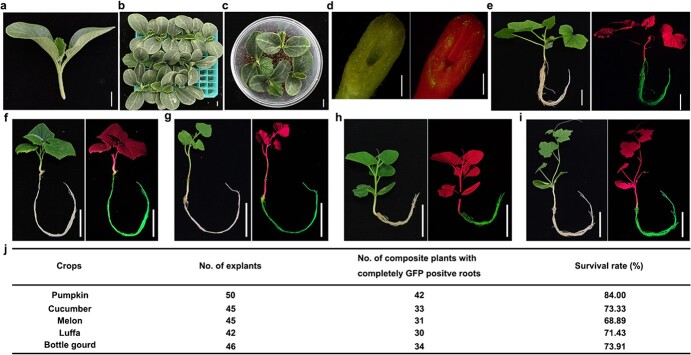
Root transformation system for cucurbit crops. **a** Pumpkin seedling explants. **b** Infection. **c** Co-cultivation. **d** GFP-fluorescence at the hypocotyl cut end after co-cultivation. **e** Composite pumpkin plants with entire roots emitting GFP fluorescence. **f–i** Composite seedlings of cucumber (**f**), melon (**g**), bottle gourd (**h**), and luffa gourd (**i**). **j** Summary of survival rates of different cucurbit crops. From (**d**) to (**i**), bright-field images (left) and UV light images (right). From (**a**) to (**c**), scale = 1 cm; (**d**), scale = 1 mm; from (**e**) to (**i**), scale = 5 cm.

## Results

### An efficient root transformation system for pumpkin and several other cucurbit crops

Constitutively expressed green fluorescent protein (GFP) was used as a reporter for transformation and to facilitate the selection of transgenic roots. The transformation was performed as shown in Supplementary Fig. S1a, with an optimized *A. rhizogenes* strain, concentration of *Agrobacterium* solution, age of seedlings for injection, and co-cultivation time (Supplementary Fig. S1b–e). In brief, hypocotyls of pumpkin seedlings whose cotyledons had just unfolded were cut at angles approximately 2 to 3 cm below the cotyledons. These excised apical parts were used as explants for transformation (Fig. 1a). The cut end of the hypocotyl was immersed in 1 mL of *A. rhizogenes* strain K599 bacterial solution containing *35S:GFP* for 30 min for infection (Fig. 1b). The infected explants were then cultivated in boxes containing sterilized vermiculite moistened with MS medium (Fig. 1c). After 4-d cultivation in the dark at 23°C, GFP fluorescence was examined. We observed that *A. rhizogenes* successfully infected the cut ends of the pumpkin hypocotyls (Fig. 1d) with an infection rate (percentage of hypocotyls with GFP fluorescence after cultivation) of approximately 95%. The explants were then transferred to trays containing sterilized vermiculite moistened with half-strength Hoagland solution. The trays were covered to maintain high humidity to promote the development of hairy roots. After 7 d, the lid was removed, roots without GFP fluorescence were excised under UV light, and the plants were returned to the vermiculite for another week. When the length of the GFP-fluorescent roots was greater than 2 cm, the plants were cultured hydroponically to more easily reveal the non-transgenic roots and allow their removal without damaging the plants. It was important to remove the non-transgenic roots at the early stages to promote the growth of transgenic roots. After the transgenic roots were fully developed, the growth of non-transgenic adventitious roots from the hypocotyl was greatly reduced. After approximately 2 weeks in hydroponic culture, healthy composite pumpkin plants with completely transgenic roots were obtained with a survival rate (percentage of healthy composite plants with completely transgenic roots) of 84.31% (Fig. 1e and j). The same root transformation method was applied to several other cucurbit crops, including cucumber, melon, bottle gourd, and luffa gourd, with survival rates of 73.33%, 68.9%, 73.9%, and 71.4%, respectively (Fig. 1f–j). Non-transformed plants were used as negative controls under UV light, as shown in Fig. S2. The morphology of the transformed pumpkin roots was similar to that of the transgene-negative roots (Fig. S3).

**Figure 2:**
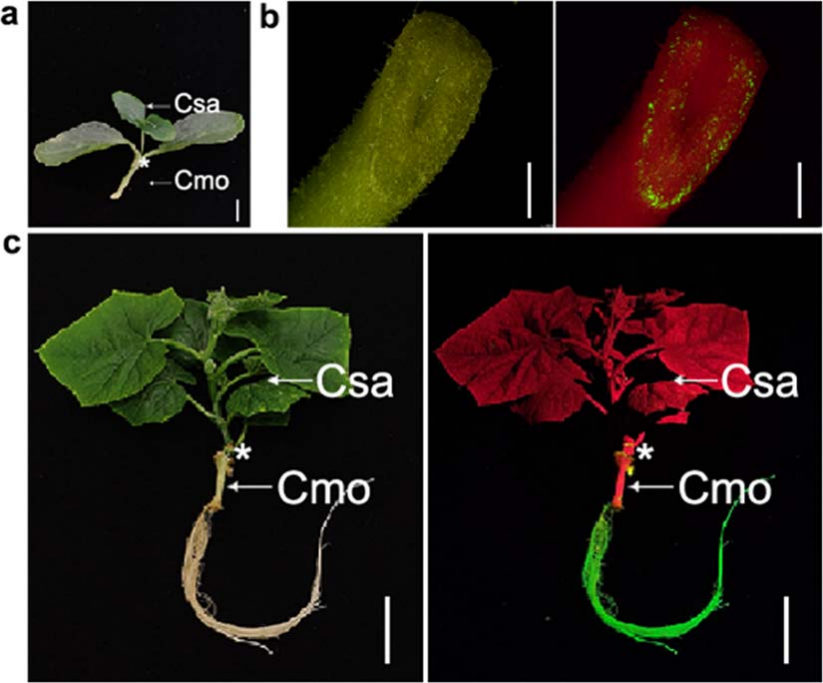
Root transformation system for cucumber/pumpkin grafts. **a** Cucumber/pumpkin grafts used as explants. **b** GFP-fluorescence at hypocotyl cut end after co-cultivation. **c** Cucumber/pumpkin grafts with entire roots emitting GFP fluorescence. Csa, cucumber; Cmo, pumpkin; *, graft junction; for (**a**) scale = 1 cm, for (**b**) scale = 1 mm, and for (**c**), scale = 5 cm.

### The root transformation system can be combined with grafting

Cucurbit crops are useful for studying grafting and graft-transmissible signals [6], and grafting a rootstock with a scion from another species is a common way to characterize mobile signals [2]. We tested whether our root transformation system could be combined with grafting, reasoning that it could provide a much needed and convenient way to genetically modify rootstocks in grafted plants. We used the cucumber/pumpkin (scion/rootstock) graft combination for our study. Pumpkin seedlings whose cotyledons had just unfolded were used as the rootstock because this stage is ideal for our *A. rhizogenes*–mediated transformation method. The pumpkin seedlings were cut at the hypocotyls as described above. Grafting was performed using the excised apical part with the hole insertion grafting method [15, 16]. Freshly made grafts were used as explants for *A. rhizogenes*–mediated transformation (Fig. 2a). The transformation and cultivation steps were performed as described above for pumpkin. After that, the cucumber/pumpkin grafts were transferred to trays containing sterilized vermiculite moistened with half-strength Hoagland solution and covered with transparent plastic lids to maintain high humidity. One week of growth in high-humidity conditions was essential, as it promoted both hairy root development and post-graft healing. For cucumber/pumpkin grafts, the infection rate of 96.1% was very similar to that of pumpkin (Fig. 2b), but the survival rate of 78.56% was lower (Fig. 2c). These results showed that the root transformation system could be combined with grafting without significantly reducing its infection and survival rates.

### *CmoHKT1;1* contributes to salinity tolerance of cucumber/pumpkin grafts by limiting long-distance Na^+^ transport

**Figure 3:**
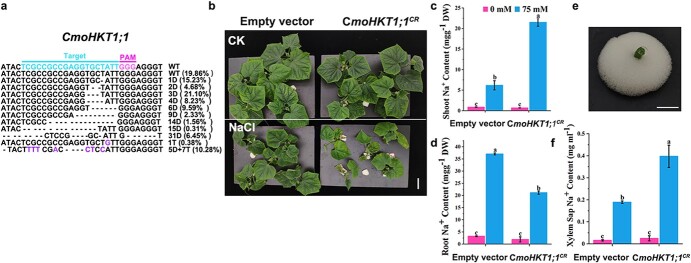
The mutation of *CmoHKT1;1* in the rootstock disturbed Na^+^ transport from the rootstock to the scion in cucumber/pumpkin grafts. **a** Editing efficiency of *CmoHKT1;1*. **b** Cucumber/pumpkin grafts transformed with *CmoHKT1;1^CR^* or an empty vector subjected to NaCl treatments, scale = 5 cm. **c–d** Na^+^ content in shoots and roots. **e** Collection of xylem sap, scale = 1 cm. **f** Na^+^ content in xylem sap. CK, 0 mM NaCl; NaCl, 75 mM NaCl. The values are mean ± standard error of three biological replicates for each treatment. Different letters indicate statistically significant differences, *P* < 0.01.

Grafting onto pumpkin has been reported to increase the salt tolerance of cucumber because the pumpkin rootstock limits the transport of Na^+^ from the roots to the shoots and thus prevents the accumulation of toxic Na^+^ in the cucumber scion [13]. The sodium transporter gene *CmoHKT1;1* plays a role in the long-distance transport of Na^+^ from roots to shoots [17]. However, the function of *CmoHKT1;1* has not been tested in pumpkin owing to the lack of an efficient and stable transformation system. In this study, we generated mutations in *CmoHKT1;1* in the pumpkin roots of cucumber/pumpkin grafts using CRISPR/Cas9 technology. The pBSE403G-sgRNA*^CmoHKT1;1^* vector was constructed by placing a *CmoHKT1;1-*targeting sgRNA in pBSE403G, which was constructed by inserting *35S:GFP* into pBSE401 [18]. *35S:GFP* was used to verify transformation. Cucumber/pumpkin grafts were transformed using *A. rhizogenes* containing pBSE403G and pBSE403G-sgRNA*^CmoHKT1;1^* vectors as described above. Four weeks after transformation, we used Hi-TOM sequencing to assess the gene-editing efficiency in the transgenic roots that were positive for GFP fluorescence [19]. The gene-editing efficiency of *CmoHKT1;1* reached 80.14% (Fig. 3a). The role of *CmoHKT1;1* was further evaluated with a 75 mM salt treatment, as described previously [17]. We determined that the *CmoHKT1;1^CR^* plants (i.e. plants transformed with pBSE403G-sgRNA*^CmoHKT1;1^*) were more sensitive to salt stress than the control plants (i.e. plants transformed with the pBSE403G empty vector) (Fig. 3b). Moreover, when subjected to salt stress, the *CmoHKT1;1^CR^* plants accumulated more Na^+^ in the shoots and less Na^+^ in the roots than the control plants (Fig. 3c and d), suggesting disturbed Na^+^ transport from roots to shoots in *CmoHKT1;1^CR^* plants. Meanwhile, when plants were subjected to salt stress, the Na^+^ content was higher in the xylem sap of the *CmoHKT1;1^CR^* plants relative to the control plants (Fig. 3e and f). These data support the conclusion that *CmoHKT1;1* contributes to salinity tolerance by limiting the long-distance transport of Na^+^ in cucumber/pumpkin grafts.

### Pumpkin *CmoNHX4* confers salinity tolerance to cucumber roots

**Figure 4:**
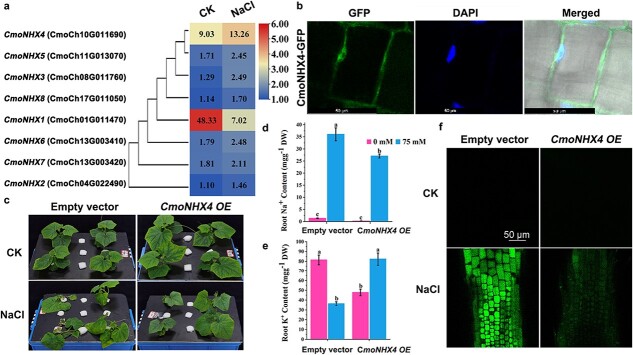
Overexpression of pumpkin *CmoNHX4* enhanced cucumber salt tolerance. **a***CmoNHX* expression; numbers indicate FPKM values. **b** Subcellular localization of *CmoNHX4-GFP*. **c** Phenotype of *CmoNHX4-OE* plants treated with NaCl. **d–e** Na^+^ and K^+^ contents in roots. **f** Na^+^ in vacuoles visualized by CoroNa staining. The values are mean ± standard error of three biological replicates for each treatment. Different letters indicate statistically significant differences, *P* < 0.01.

We also tested whether our method could be used for cell biological analyses in the roots of cucurbit crops. The higher salt tolerance of pumpkin relative to cucumber is associated with the higher K^+^ uptake capacity of pumpkin [5]. The tonoplast Na^+^/H^+^ antiporter *AtNHX1* mediates potassium uptake [20]. In this study, we observed that the expression of *CmoNHX4*—a homolog of *AtNHX1*—was induced by NaCl treatment, and it was the most highly expressed member of the *CmoNHX* gene family in pumpkin roots treated with NaCl (Fig. 4a and Fig. S4). We therefore tested whether the overexpression of *CmoNHX4* in cucumber could improve salt tolerance. Vectors were constructed by inserting the *CmoNHX4* coding sequence with or without a GFP tag downstream of the 35S promoter (Fig. S5a,b). *35S:DsRed2* was used to monitor transformation [21]. The vectors were transformed as described above. The *35S:CmoNHX4-GFP* vector was used for the subcellular localization of CmoNHX4-GFP. The expression of CmoNHX4-GFP in cucumber roots was low and inconsistent (Fig. S6a,b), similar to 35S:AtNHX1-GFP expression in the roots of transgenic *Arabidopsis* [22]. According to a previous report [22], the GFP signal within the vacuolar lumen and endosomes in cells with bright GFP expression may be the result of 35S promoter–driven overexpression (Fig. S6a,b). However, the CmoNHX4-GFP signal was found on the vacuolar membrane in cells with lower GFP signal, as evidenced by nuclei stained with DAPI detected outside the membrane boundary (Fig. 4b). The expression pattern of 35S:CmoNHX4-GFP in pumpkin roots was similar to that in cucumber roots, but the fluorescence intensity in pumpkin roots was lower (Fig. S6c–e). The intensity of the GFP signal diminished after plasmolysis of root cells induced by sucrose, with the vacuolar membrane–located GFP signal barely detectable and only a GFP signal within the vacuolar lumen or endosomal bodies observed outside the nuclei (Fig. S6f). The tonoplast membrane marker *35S:AtTIP1-RFP* was then transiently co-expressed with *35S:CmoNHX4-GFP* in *Nicotiana* leaves [23]. The colocalization of CmoNHX4-GFP with AtTIP1-RFP provided additional evidence that CmoNHX4-GFP was localized on the vacuolar membrane (Fig. S6g). The *35S:CmoNHX4* vector (without GFP tag) was used to overexpress *CmoNHX4*, and the resulting cucumber seedlings with transformed roots were used for phenotypic analysis and CoroNa green staining. Real-time PCR analysis showed that *CmoNHX4* expression increased significantly in roots transformed with the *35S:CmoNHX4* vector compared with the empty vector (Fig. S5c). The overexpression of *CmoNHX4* in cucumber roots significantly improved the salt tolerance of cucumber (Fig. 4c). We measured the Na^+^ and K^+^ content in control and *CmoNHX4*-overexpressing roots and found that *CmoNHX4* overexpression decreased the Na^+^ content and increased the K^+^ content of cucumber roots (Fig. 4d and e). We used CoroNa staining [24] to assess the accumulation of Na^+^ in the vacuoles of cucumber roots and observed that the *CmoNHX4*-overexpressing roots accumulated less Na^+^ in their vacuoles than the control roots (Fig. 4f). These data indicate that *CmoNHX4* conferred salinity tolerance to cucumber plants by increasing the K^+^ content and decreasing the Na^+^ content of cucumber roots.

In conclusion, we developed a robust root transformation system for cucurbit crops. We obtained composite plants with transgenic roots using the apical parts of seedlings as explants. A hydroponic culture procedure allowed us to readily identify non-transgenic roots and excise them without damaging the plants. By combining our transformation system with grafting, we provide a promising approach for studying shoot–root communication. Using this system, we further explored the mechanism of higher salt tolerance in pumpkin compared with cucumber by revealing the roles of *CmoHKT1;1* and *CmoNHX4*.

## Discussion

Stable genetic transformation mediated by *Agrobacterium tumefaciens* is time-consuming, labor-intensive, and a major constraint in the functional genomics of cucurbit crops. In addition, the recalcitrant nature of some cucurbits reduces their transformation efficiencies, and an alternative technique is required to address these issues [1, 25]. Hairy root transformation has been successfully performed in hundreds of plant species [26–28], including legumes like soybean [11] and peanuts [29], vegetables like potato [30] and tomato [31], and even fruits such as avocado [32], grapevine [33], and *Prunus* [34]. The hairy root transformation system has been used in biological research for decades and has proven to be a viable tool for studying gene function.

In the current study, we successfully developed a hairy root transgenic system that may be deployed to investigate gene function in cucurbit crops. This approach highlights the basic need for genetic verification systems, particularly in pumpkin, melon, bottle gourd, and luffa gourd, which currently lack highly efficient stable transgenic systems. This root transformation system, together with stably transformed roots, could be used for gene functional studies in the roots of cucurbit crops using overexpression and knockout techniques, promoting research in the fields of root metabolites, nutrient acquisition, root development, and root resistance to soil-borne disease and abiotic stress. It could also be used for cell biological analyses in roots, such as subcellular localization and cell staining. Moreover, this root transformation system was used in combination with grafting to explore root–shoot communication in grafted cucurbit plants. However, there were a few limitations, such as the inability to obtain homozygous mutations edited by CRISPR/Cas9 and the fact that the resulting mutations could not be transmitted to the next generation. Nevertheless, its convenience, speed, and high efficiency make this system a promising tool for the preliminary validation of candidate genes before performing stable transformation. Our method of studying scion–rootstock communication by integrating the root transformation system with a grafting technique could be used in other crops such as flowers and fruit trees.

## Materials and methods

### Plant materials and growth conditions

Pumpkin (*Cucurbita maxima* × *Cucurbita moschata*, Fenglejinjia), cucumber (*C. sativus* L., Jinchun No. 4), melon (*C. melo*, Yilishabai), bottle gourd (*L. siceraria*, Jingxinzhen No.1), and luffa gourd (*L. acutangular*, Zaojia) were used in this study. The seedlings were grown in a climate chamber with a light intensity of 350 μmol·m^−2^·s^−1^, a photoperiod of 16 h light/8 h dark, and day/night temperatures of 28°C/18°C.

### Vector construction

The pBSE403G vector was constructed by inserting *35S:GFP:Terminator* into the *Eco*RI site of pBSE401 [18]. The sgRNA targeting *CmoHKT1;1* was designed using Geneious software as described previously [8]. The *CmoHKT1;1*-targeting sgRNA was cloned into pBSE403G as described previously [18]. For the overexpression of *CmoNHX4*, the intermediate vector pBSE401R was first constructed by inserting *35S:DsRed2:Terminator* [21] into the *Eco*RI site of the pBSE401 vector. Then, *CmoNHX4* or *CmoNHX4*-*GFP* was cloned into pBSE401R between the *Xba*I/*Sac*I sites and downstream of the 35S promoter. The pBSE401R-CmoNHX4 vector was used for phenotypic analysis and CoroNa green staining. pBSE401R-CmoNHX4-GFP was used to study the subcellular localization of *CmoNHX4*. Maps of the two vectors are provided in Fig. S5.

### *A. rhizogenes*–mediated root transformation system

Seeds were soaked in water at 55°C for 5 h and then incubated in a Petri dish at 28°C in the dark until germination. *A. rhizogenes* strain K599 from cultures with OD_600_ values between 0.8 and 1.0 were collected and re-suspended in MS medium containing 200 μM AS for bacterial infection. The subsequent steps are described in detail in the supplementary data. The hole insertion grafting method was used for grafting [15, 16]. In brief, we removed the apical meristem of the rootstock, leaving the cotyledons, and made a hole on the top portion with a grafting needle. Then, the cucumber scion was cut at angles on both sides of the hypocotyls and inserted into the hole made in the rootstock.

### DNA isolation and Hi-TOM analysis

About 2 weeks after co-cultivation, healthy composite plants with completely transgenic roots were used to analyze the efficiency of gene editing. The GFP-positive transgenic roots were used for DNA extraction with the CTAB protocol. The fragment containing the target site was PCR amplified and analyzed by Hi-TOM sequencing [19].

### Hydroponic cultivation and salt treatment

For hydroponic culture, plants were grown in half-strength Hoagland solution in plastic boxes (380 mm × 245 mm × 100 mm) as described previously [17]. Foam boards were used to anchor and support the plants. The Hoagland solution was renewed once a week. For the salt treatment, healthy plants with completely transgenic GFP or DsRed2 fluorescent roots and consistent growth were selected and grown in 5 L half-strength Hoagland solution containing 0 or 75 mM NaCl for 5 days. Three biological replicates per treatment were used for the experiment.

### Measurements of Na^+^ and K^+^ content

Three plants per treatment were harvested. The roots and shoots were separated and placed into Kraft paper bags, which were then placed at 105°C for 15 min and 80°C until dry. The dried samples were ground into powder and weighed. Then, 0.1 g of powder was digested in 5 mL H_2_SO_4_ at 300°C for 1 h, and a drop of H_2_O_2_ was added every half hour until the solution became colorless and transparent. The digested solution was diluted and used to determine the concentrations of Na^+^ and K^+^ using an atomic absorption spectrophotometer (6300C, Shimadzu). For the determination of Na^+^ concentration in xylem sap, 20 μL xylem sap was diluted in 5 mL ddH_2_O and directly measured without digestion. Three biological replicates per treatment were used for the experiment.

### Gene expression analysis by qRT-PCR

Cucumber roots transformed with an empty vector or a pBSE401R-CmoNHX4 vector were harvested and immediately frozen in liquid nitrogen for gene expression analysis. *CmoNHX4* expression was measured according to a previously described method [35]. Primer3Plus software was used to generate specific primer sequences as listed in supplementary Table S1.

### Xylem sap collection

Cross sections of the salt-treated and control plants were cut 1 cm above the graft union. The wound surface was then wiped with filter paper two to three times, and the xylem fluid, which flowed out because of root pressure, was carefully removed with a pipette and placed into a 1.5-mL centrifuge tube for subsequent ion determination.

### Light microscopy and CoroNa staining

Na^+^ content in pumpkin root vacuoles was measured with the green fluorescent Na^+^ dye CoroNa as described previously [24]. Transgenic roots positive for *35S:DsRed2* were used for this analysis. The DMSO-dissolved dye was diluted to 20 μM with the measuring buffer (10 mM KCl, 5 mM Ca^2+^-MES, pH 6.1). Excised root tip segments (~1 cm) were immersed and incubated in the dark for 2.5 h for staining. The stained roots were washed three times with measuring buffer and then observed with a confocal laser scanning microscope (SP8; Leica Microsystems). Co-localization of CmoNHX4-GFP and AtTIP-RFP was evaluated in tobacco leaves as described previously [17]. The nuclei were stained with 10 μg/mL DAPI solution for 10 min.

## Acknowledgements

This research was supported by the National Key Research and Development Program (2018YFD100800), the National Natural Science Foundation of China (32072653, 31772357, 31801866), the Natural Science Foundation of Hubei Province (2019CFA017), the Fundamental Research Funds for the Central Universities (2662018PY039, 2662018QD021), and the China Agriculture Research System of MOF and MORA (CARS-25). We thank Prof. Robert Larkin for critical reading of our manuscript.

## Author contributions

L.Y. and Z.B. conceived and designed the experiments. S.G., H.S., H.C., J.S., Z.C., L.Z., W.W., and R.Y. performed the experiments and analyzed the data. L.Y. and Z.B. wrote the paper.

## Data availability

All the data generated in this study are included in the published article and its supplementary information.

## Conflict of interest

The authors declare that they have no competing interests.

## Supplementary data

Supplementary data is available at *Horticulture Research Journal* online.

## Supplementary Material

Web_Material_uhab082
