# Supplementary material for: An efficient root transformation system for CRISPR/Cas9-based analyses of shoot–root communication in cucurbit crops
Source: Hortic Res. 2022 Jan 20;9:uhab082. doi: 10.1093/hr/uhab082 (PMC9071382; doi:10.1093/hr/uhab082)
Supplement: Web_Material_uhab082 [file web_material_uhab082.docx]

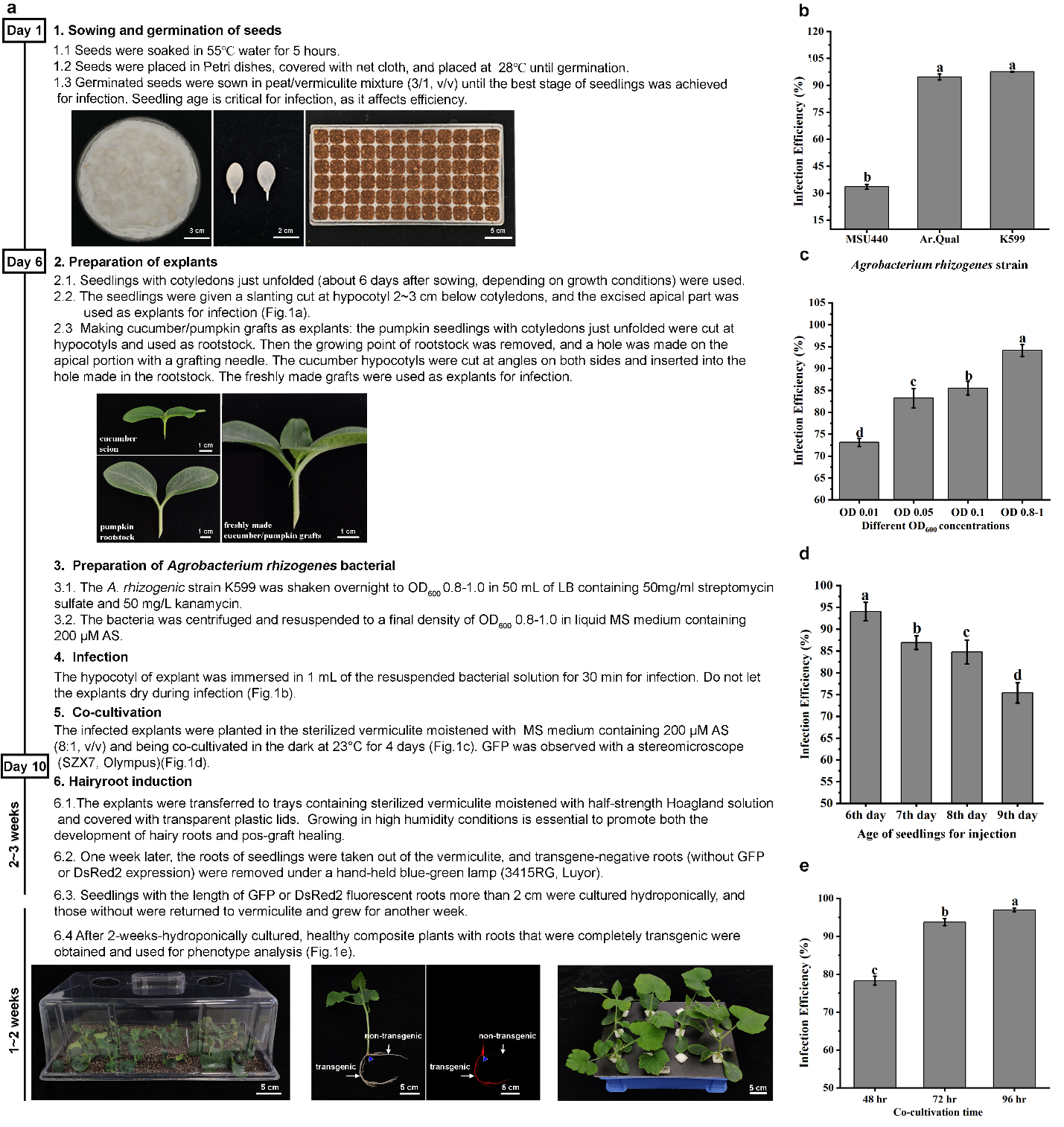


**Fig. S1** Summary of root transformation procedure for cucurbit crops and cucumber/pumpkin graft combination.

**a** Procedure of the *Agrobacterium rhizogenes*-mediated root transformation system. Effect of *A. rhizogenes* strain **b,** the concentration of *Agrobacterium* solution **c,** age of seedlings for infection **d,** and the co-cultivation time **e,** on *Agrobacterium* infection efficiency. Values are means +SE, n = 3. Different letters indicate statistically significant differences, *P*<0.01.


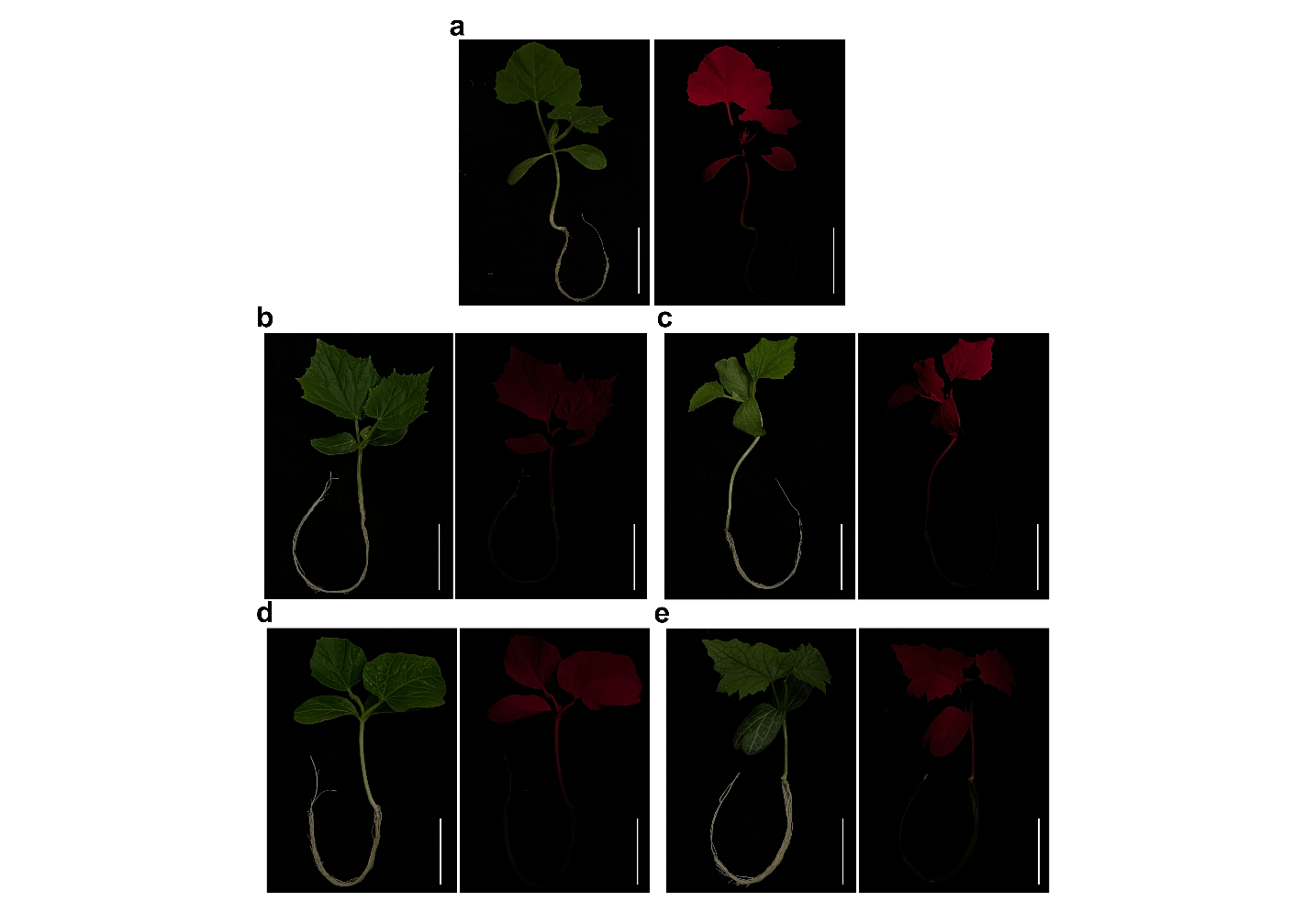


**Fig. S2** Non-transformed seedlings of cucurbitaceae plant**.**

**a** pumpkin. **b** cucumber. **c** melon. **d** bottle gourd. **e** luffa gourd. Non-transformed plants under bright-field images (left) and UV light images (right). Scale = 5 cm.


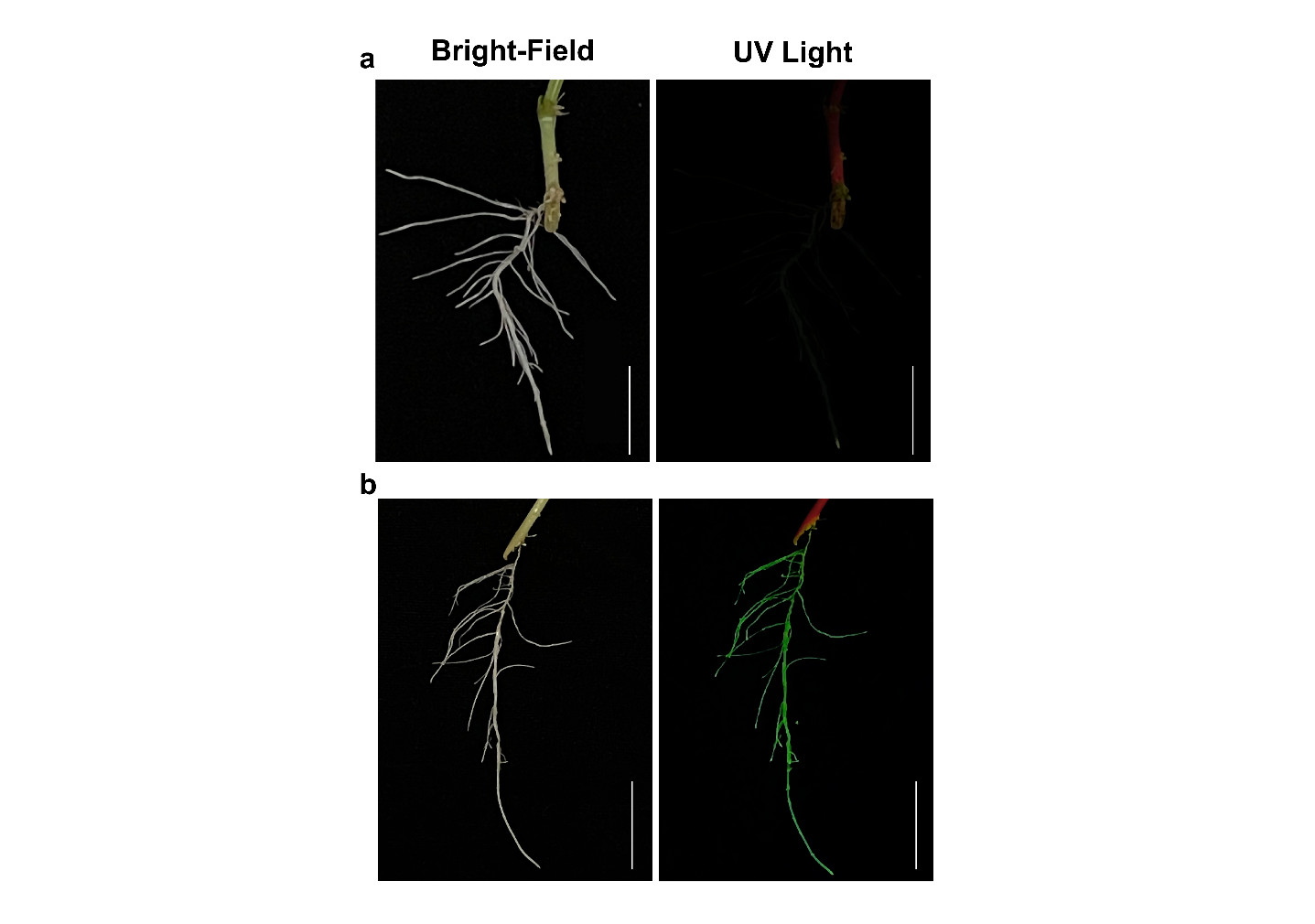


**Fig. S3** The morphology of transgene-positive and transgene-negative pumpkin roots.

The morphology of **a** transgene-negative and **b** transgene-positive roots of pumpkin. Scale = 2 cm.


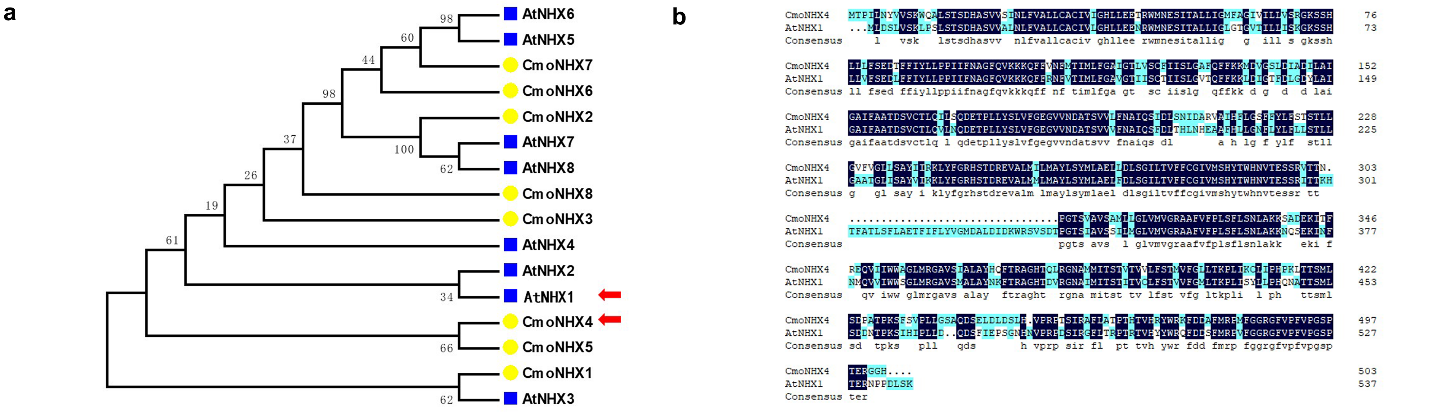


**Fig. S4** Phylogenetic relationship of CmoNHXs with *Arabidopsis thaliana* NHXs.

**a** Phylogenetic tree of CmoNHXs and AtNHXs. **b** CmoNHX4 has the highest homology with AtNHX1, with a protein sequence homology rate of 71.86%. Alignment of CmoNHX4 and AtNHX1were presented; identical and similar amino acids are represented by dark blue and light blue, respectively. Phylogenetic analyses were conducted in MEGA7. Sequences were aligned using DNAMAN (Lynnon Biosoft).


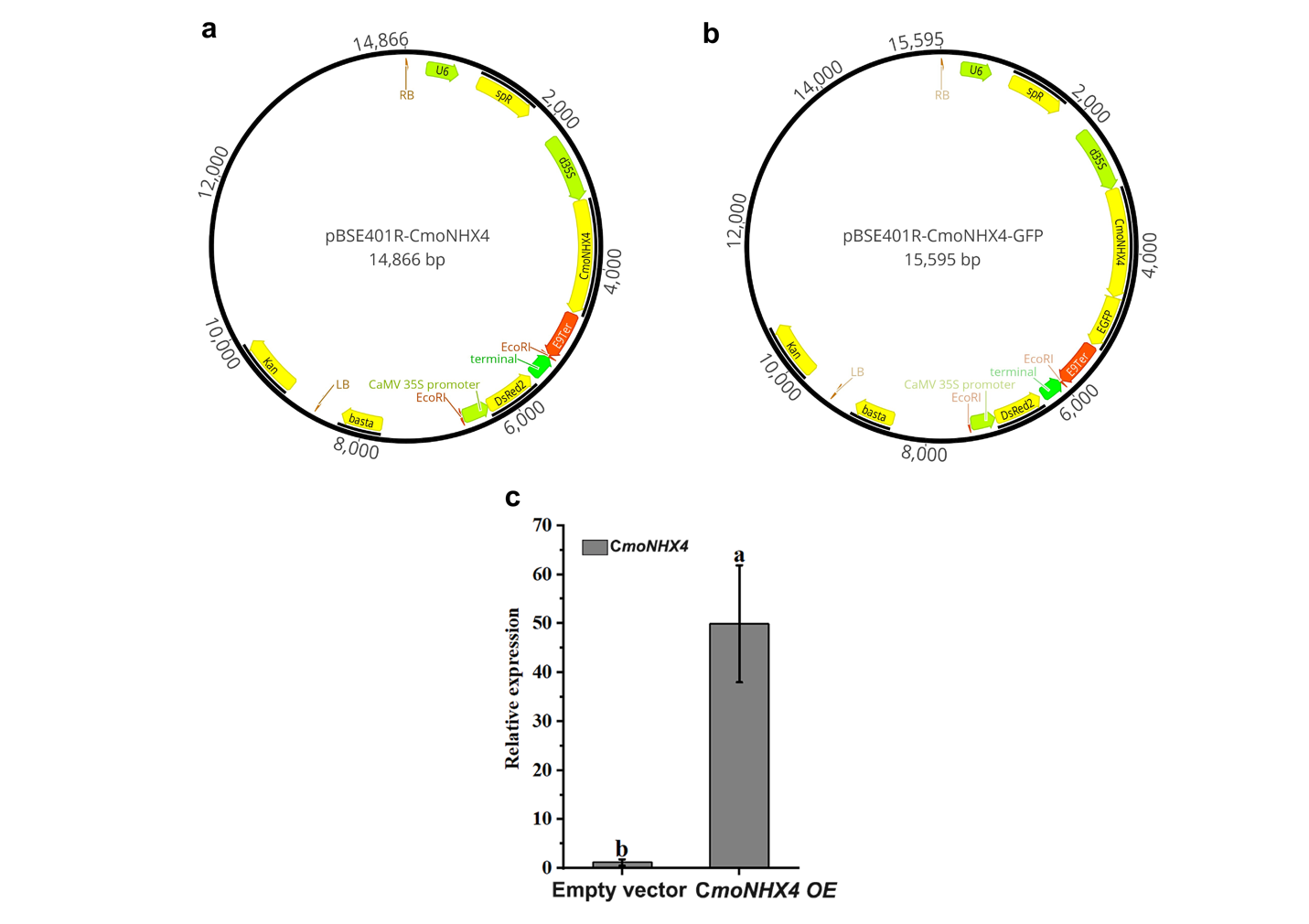


**Fig. S5** Vector maps and *CmoNHX4* expression analysis by qRT-PCR.

**a** Vector map of *CmoNHX4 OE* construct used for overexpression. **b** Vector map of *CmoNHX4-GFP* construct used for subcellular localization experiment. **c** qRT-PCR detected the expression of *CmoNHX4* in cucumber roots transformed with empty vector and *CmoNHX4* OE vector. Error bars show the SE of the values from six replicates. Different letters indicate statistically significant differences, *P*<0.01.


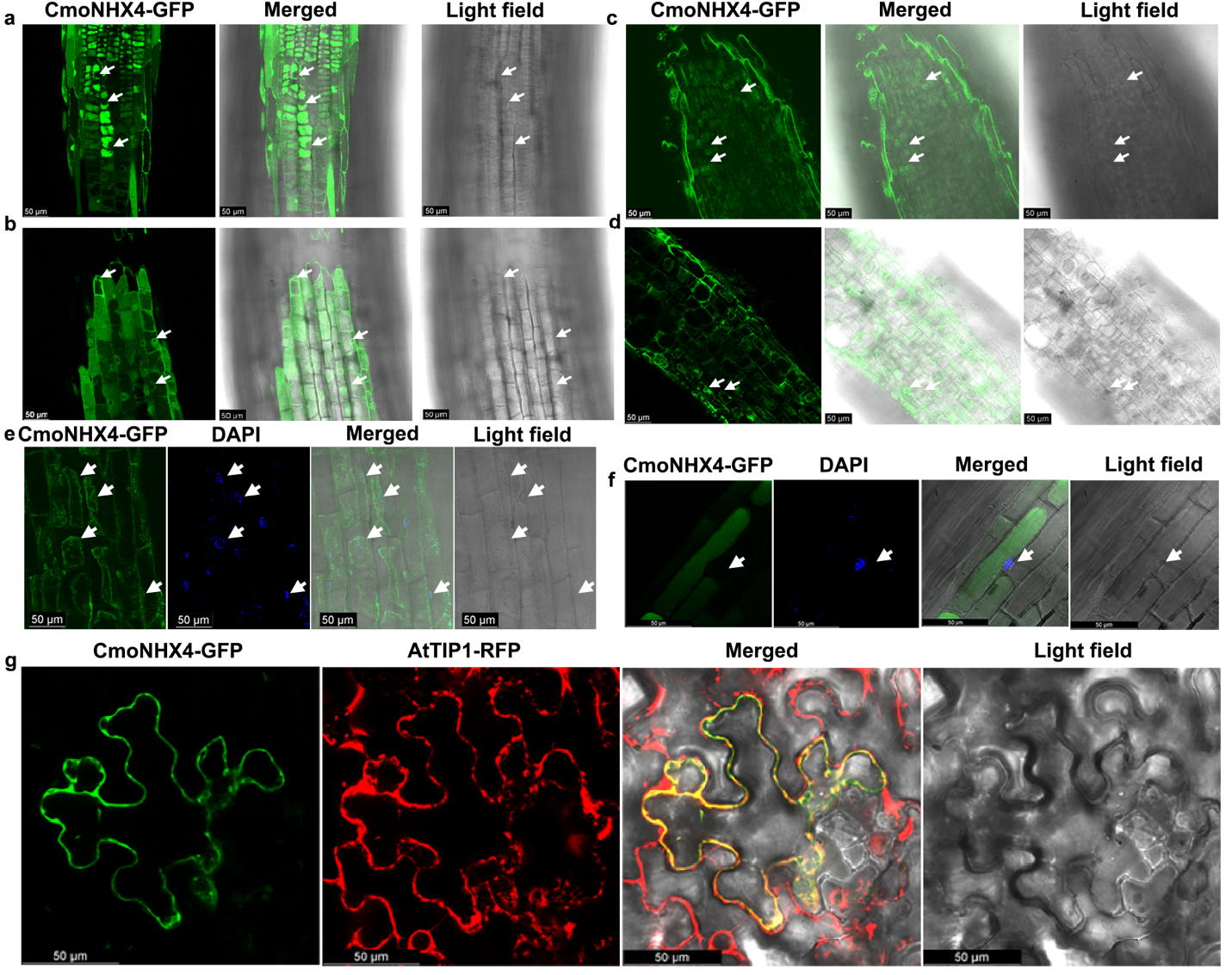


**Fig. S6** Subcellular localization of *CmoNHX4*

**a** CmoNHX4-GFP fluorescence in cells near the cucumber root tip and **b** in the elongation zone. **c** CmoNHX4-GFP fluorescence in pumpkin root tip cells and **d** in the elongation zone cells. **e** The vacuolar membrane location of CmoNHX4-GFP was shown with DAPI stained the nuclei in pumpkin root cells. **f** The vacuolar lumen location of CmoNHX4-GFP was shown with DAPI stained the nuclei in cucumber root cells after plasmolysis. **g** Co-localization of CmoNHX4 and the tonoplast membrane marker 35S:AtTIP1-RFP by transient expression in tobacco epidermal cells. Arrows point to nuclei.

**Table. S1** Primer sequences used for qRT-PCR

| Primer Name | Sequence |
| --- | --- |
| CmoNHX4-qPCR F | GAGTTACCACCAATCCGGGG |
| CmoNHX4-qPCR R | GTAGTTGAGTGTGCCCTGCT |
| CmoActin F | CTGGACTCTGGTGATGGTGT |
| CmoActin R | CGTTCAGCAGTGGTTGTGAA |
